# Prevalence and types of anemia among people with tuberculosis in Africa: a systematic review and meta-analysis

**DOI:** 10.1038/s41598-023-32609-1

**Published:** 2023-04-03

**Authors:** Yeshewas Abaynew, Ahmed Ali, Girma Taye, Melese Shenkut

**Affiliations:** 1 Department of Biostatistics and Epidemiology, College of Medicine and Health Sciences, Wollo University, Dessie, Ethiopia; 2 Department of Preventive Medicine, School of Public Health, College of Health Sciences, Addis Ababa University, Addis Ababa, Ethiopia; 3 Department of Anatomy, College of Medicine and Health Sciences, Wollo University, Dessie, Ethiopia

**Keywords:** Diseases, Medical research

## Abstract

Globally, tuberculosis (TB) and anemia are public health problems related with high morbidity and mortality. Furthermore, anemia is frequently manifested among people with TB in Africa, prevalence ranging from 25 to 99%. The presence of anemia is associated with an increase in individuals’ susceptibility to TB and poor treatment outcomes. Studies have reported heterogeneous estimate of prevalence of anemia among people with TB in Africa. This review aimed to estimate the prevalence of anemia among newly diagnosed people with TB n Africa. We searched studies in Medline/PubMed, Cochrane library, ScienceDirect, JBI database, the Web of Science, Google Scholar, WorldCat, Open Grey, Scopus, Agency for Healthcare Research and Quality, ProQuest, and African Journals Online that reported the prevalence of anemia at TB diagnosis. Two reviewers performed data extraction with pre-defined inclusion criteria. A random-effects logistic regression model was used to pool the prevalence of anemia and levels of anemia with a 95% confidence interval (CI) in STATA version 14. Heterogeneity and publication biases were explored. A total of 1408 studies were initially identified, and seventeen studies with 4555 people with TB were included in the analysis. The prevalence of anemia among people with TB in Africa was 69% (95% CI 60.57–77.51). The pooled prevalence of anemia of chronic disease was 48% (95% CI 13.31–82.75) and normocytic normochromic anemia was 32% (95% CI 13.74–50.94) while mild anemia was 34% (95% CI 20.44–46.86). Females were more anemic than males at TB diagnosis in Africa (74% vs. 66%). The finding indicates that anemia is a common co-morbidity present among people with TB, especially among females. Mild anemia and normocytic normochromic anemia were more common at TB diagnosis. The finding indicates that anemia is a common co-morbidity present among people with TB in Africa region. Hence, it is recommended to instigate a routine anemia screening at TB diagnosis to improve treatment outcomes.

## Introduction

Tuberculosis (TB) is principally caused by the bacillus *Mycobacterium tuberculosis* and can manifest as either pulmonary or extra-pulmonary TB^1^. Globally, a total of 1.6 million deaths recorded and an estimated 10.6 million people have developed TB in 2021^1^.

Globally, anemia is a worldwide public health problem^2^ that affects one-quarter of the world’s population with an estimated global prevalence of 24.8% in 2008^3^. In 2010, Kassebaum et al.^4^ affirmed that 32.9% of the world population were anemic. Globally in 2019, the prevalence of anaemia in non-pregnant women aged 15–49 years 30%, while in pregnant women aged 15–49 years it 36% (34–39)^5^.

Anemia is functionally defined as insufficiency of erythrocyte mass to deliver oxygen in sufficient amount to peripheral tissues^6^. The effects of anemia is diverse among people with TB such as a risk factor for the development of TB^7^ and is associated with TB complications including lung injury and poor prognosis such as poor sputum conversion 2 months after TB treatment initiation and also an increased risk of deaths^8–11^.

Anemia among people with TB has been related to inflammatory mediators on erythropoiesis^12^, iron-deficiency^9,12^, chronic inflammation^13^, the disease itself^14^, hemoptysis, malnutrition^6^, bone marrow suppression, and failure of iron utilization^15,16^. Chronic inflammation and iron deficiency are predominant contributors to the presence of anemia among people with TB^9^.

Studies in Africa have reported anemia to be the most common hematological derangement among people with TB, however, the prevalence of anemia at TB diagnosis vary widely ranging from 25 to 99%^17,18^. Existing studies are heterogeneous due to variation in the sample size, methods, population characteristics, and definitions of anemia. Available studies used different criteria to define anemia including the WHO criteria. Additionally, the small sample size among studies might contribute to the varied estimate of prevalence of anemia. Furthermore, the variation in prevalence of anemia among studies may be attributed to the inclusion of TB-HIV co-infected people in some studies since HIV infected people hemoglobin levels are significantly lower than HIV-negative people^19^.

Multiple studies have also documented the types of anemia presented at TB diagnosis, but findings are inconsistent across studies; in many studies, mild anemia has been commonly encountered at TB diagnosis^18,20^. Yet, other studies have reported moderate anemia^21–24^. Conversely, severe anemia is relatively a rare event with prevalence ranges between 2.5 and 32.5%^17,25^. This inconsistency of the prevalence of the types of anemia among people with TB could be attributed to variations of studies in terms of the sample size and study population.

Morphologically, studies have indicated that normocytic normochromic anemia is commonly present at the time of TB diagnosis^20,22^. However, in some studies microcytic hypochromic anemia is profoundly encountered at TB diagnosis^18,24^. Moreover, macrocytic anemia is often identified in less than 10% of patients at the time of diagnosis of TB^18^. Normocytic hypochromic picture is also reported in studies with a variable frequency, including 32.5% in Ethiopia^23^, 47.5% in the Democratic Republic of Congo^24^. It is noted that the observed variation might be associated to the impact of HIV/AIDS and the differences in the cutoff values used to define the morphological pattern of anemia.

The heterogeneity in the prevalence of anemia among people with TB at diagnosis prompted us to consider a systematic review and meta-analysis. In addition, there is no data on the estimate of the prevalence and level of anemia among people with TB in Africa. The review was conducted to estimate the prevalence and levels of anemia including the morphological patterns at TB diagnosis that is needed to consider anemia co-morbidity the management of TB.

Anemia is an important co-morbid condition among people with TB and has been associated with poor prognosis during treatment. So, there is a need to know the characteristic of anemia as a risk factor associated with poor complications of TB to institute an intervention to address anemia among people with TB to achieve the END TB strategy.

## Results

### Description of studies

**Figure 1:**
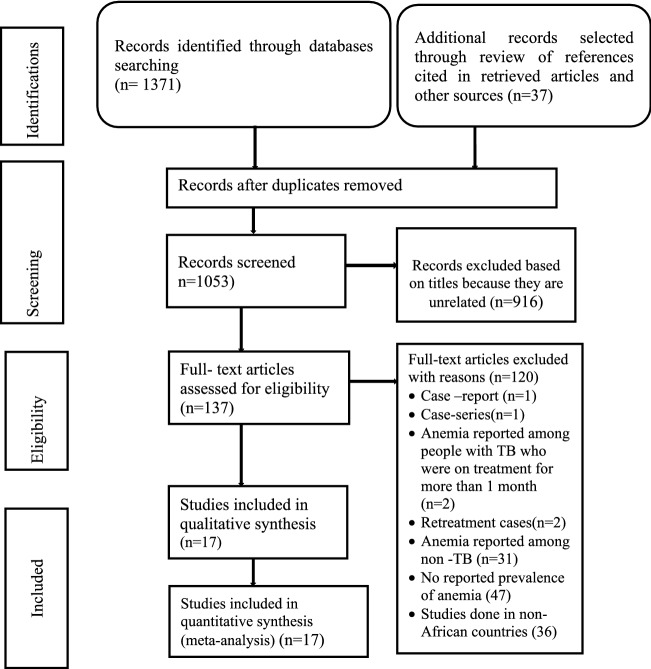
Preferred reporting items for systematic reviews and meta-analyses (PRISMA)^26^ flow diagram indicating the selection of studies for the systematic review and meta-analysis.

As indicated in Fig. 1, the searching in electronic databases identified 1371 studies, and 37 studies were manually included with review of references cited in the retrieved studies. A total of 355 studies were duplicated and were removed. Moreover, 916 studies were unrelated to the purpose of the current review and were excluded from further review process. The remaining 137 studies were selected to undergo a full paper review (Fig. 1). Finally, the review was done on 17 studies that scored 5 and above on the Joanna Briggs Institute (JBI) quality appraisal criteria.

### Characteristics of included studies

As depicted in Table 1, a total of 11 cross-sectional studies, 5 case–control studies, and one randomized clinical trial were included. Among studies included, one was from Democratic Republic of Congo^24^, another was from Egypt^18^, four were from Ethiopia^17,20,23,27^, one was from Gambia^28^, two were from Malawi^15,29^, one was from Nigeria^30^, one was from South Africa^25^, two were from Sudan^22,31^, and four were from Tanzania^9,21,32,33^. Regarding the sample size of included studies, 39 is the smallest number of participants^28^ and 1245 is the maximum number of participants^21^ (Table 1).

**Table 1:** A description of studies included in the systematic review and meta-analysis, 2021.

No.	Authors, year, country	Study design	Sample size	Males	Types of TB	Anemic patients	Anemic males	Anemic females	Quality score
1	Mohammed, 2016^22^, Sudan	Case–control study	40	25	SPPTB/HIV−	34	22	12	5/10
2	Isanaka et al., 2012^9^, Tanzania	Randomized, placebo-controlled trial	684	448	TB/HIV+	438	240	198	8/13
3	Abdelkareem et al., 2015^18^, Egypt	Cross-sectional descriptive study	100	42	PTB/HIV−	100			7/9
4	Atomsa et al., 2014^23^, Ethiopia	Comparative cross-sectional study	108	45	TB	40			5/9
5	Gunda et al., 2014^32^, Tanzania	Retrospective cross-sectional study	701	361	PTB/HIV+	358			7/9
6	van Lettow et al., 2005^15^, Malawi	Cross-sectional study	500	227	SPPTB/HIV+	427			8/9
7	Abay et al., 2018^27^, Ethiopia	Comparative Cross-Sectional Study	100	51	PTB/HIV+	53			7/9
8	Bashir et al., 2015^31^, Sudan	Case–control study	100	77	SPPTB/HIV−	44			5/10
9	Mulenga et al., 2017^24^, Democratic Republic of Congo	Prospective cross-sectional study	200	130	SPPTB/HIV+	139			6/9
10	Erhabor et al., 2020^30^, Nigeria	Case–control study	80	67	SPPTB/HIV−	71			5/10
11	Minchella et al., 2015^28^, Gambia	Comparative cross-sectional study	39	28	PTB/HIV+	26			7/9
12	Kerkhoff et al., 2016^25^, South Africa	Comparative cross-sectional study	153	53	TB/HIV+	131	44	87	7/9
13	Kahase et al., 2020^17^, Ethiopia	Comparative cross-sectional study	40	25	PTB/HIV−	10			8/9
14	Hella et al., 2018^33^, Tanzania	Case–control study	102	78	SPPTB/HIV+	74			9/10
15	Yesuf, 2017^20^, Ethiopia	Case–control study	44	30	TB/HIV+	30			6/10
16	Nagu et al., 2014^21^, Tanzania	Prospective cross-sectional study	1245	831	SPPTB/HIV+	1067			7/9
17	van Lettow et al., 2004^29^, Malawi	Cross-sectional study	319	149	SPPTB ± HIV	270	124	146	7/9

### Prevalence of anemia among people with tuberculosis

**Figure 2:**
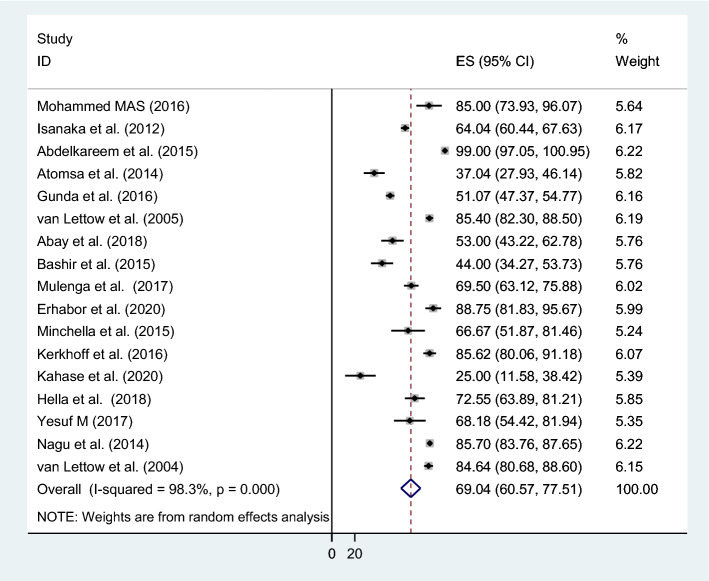
The pooled prevalence of anemia among people with TB.

Among 4555 people with TB included in the review, 3311(73%) had anemia. A total of 17 studies were found to be eligible to calculate the overall prevalence of anemia among people with TB, and the pooled prevalence of anemia was 69% (95% CI 60.57–77.51; I^2^ = 98%, p = 0.000) (Fig. 2).

**Figure 3:**
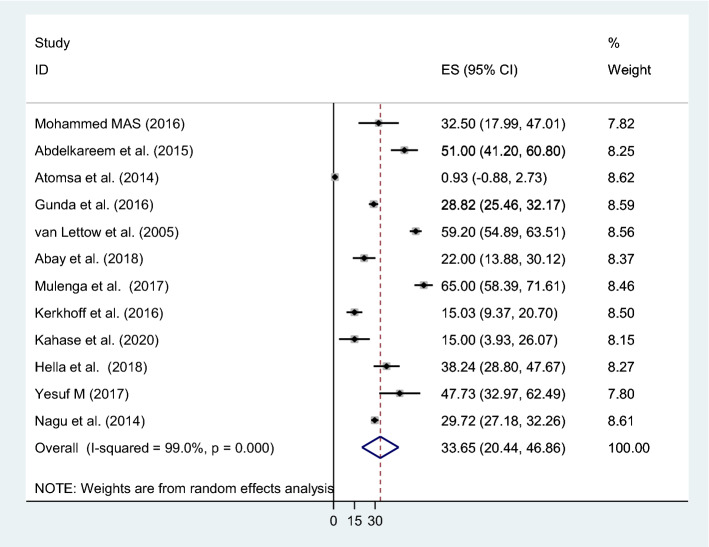
The pooled prevalence of mild anemia in patients with TB.

The pooled estimate of mild, moderate and severe anemia was 34% (95% CI 20.44–46.86; I^2^ = 99%, p = 0.000), 29% (95% CI 15.81–43.12; I^2^ = 99%, p = 0.000) and 10% (95% CI 5.77–14.28; I^2^ = 95%, p = 0.000) respectively (Fig. 3).

**Figure 4:**
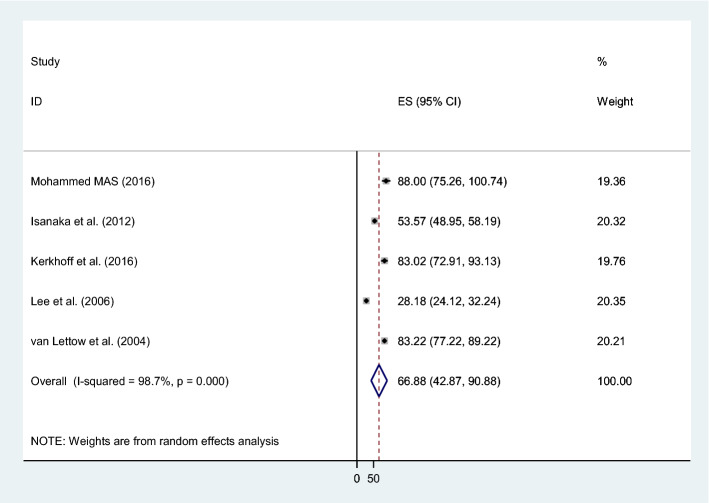
The pooled prevalence of anemia among male TB patients.

**Figure 5:**
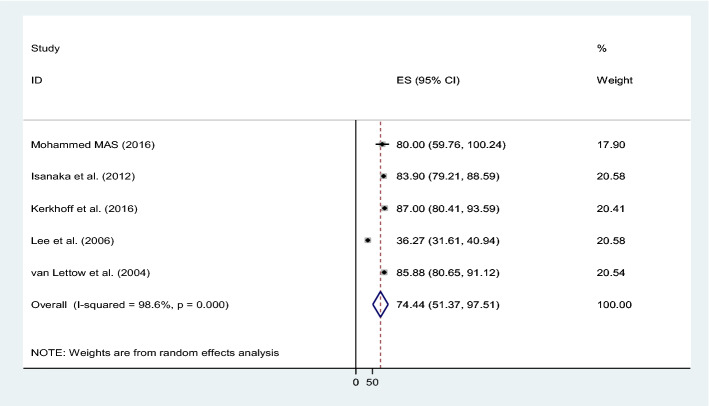
The pooled prevalence of anemia among female population with TB patients.

The combined prevalence of anemia in male and female patients was 67% (95% CI 42.87–90.88; I^2^ = 99%, p = 0.000) and 74% (95% CI 51.37–97.51; I^2^ = 99%, p = 0.000) respectively (Figs. 4 and 5).

### Types of anemia presented at the time of TB diagnosis

**Figure 6:**
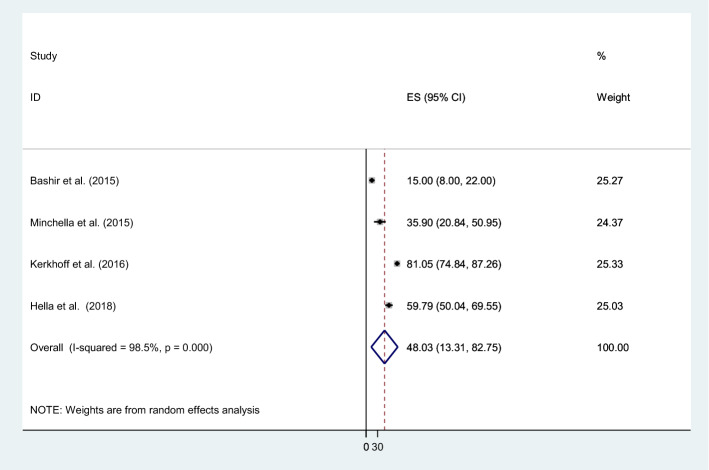
The pooled prevalence of anemia of chronic disease at the time of TB diagnosis.

Anemia of chronic disease was the most common type of anemia identified in TB, 48% (95% CI 35.6–74.99; I^2^ = 98%, p = 0.000) followed by iron deficiency anemia, 11% (95% CI 2.43–19.90; I^2^ = 99%%, p = 0.000) (Fig. 6).

### Morphological patterns of anemia among people with tuberculosis

**Figure 7:**
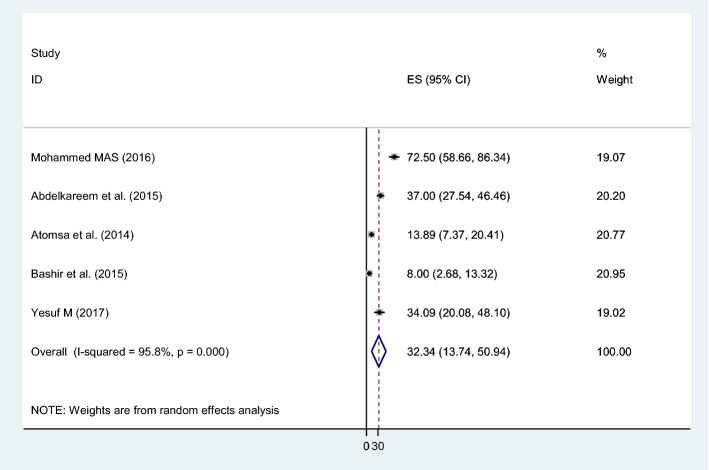
The pooled prevalence of normocytic normochromic anemia at TB diagnosis.

Considering the morphological patterns of anemia at TB diagnosis, the pooled prevalence of normocytic normochromic blood picture was 32% (95% CI 13.74–50.94; I^2^ = 96%, p = 0.000), and microcytic hypochromic anemia was 26% (95% CI 8.20–43.53; I^2^ = 96%, p = 0.000) (Fig. 7).

**Figure 8:**
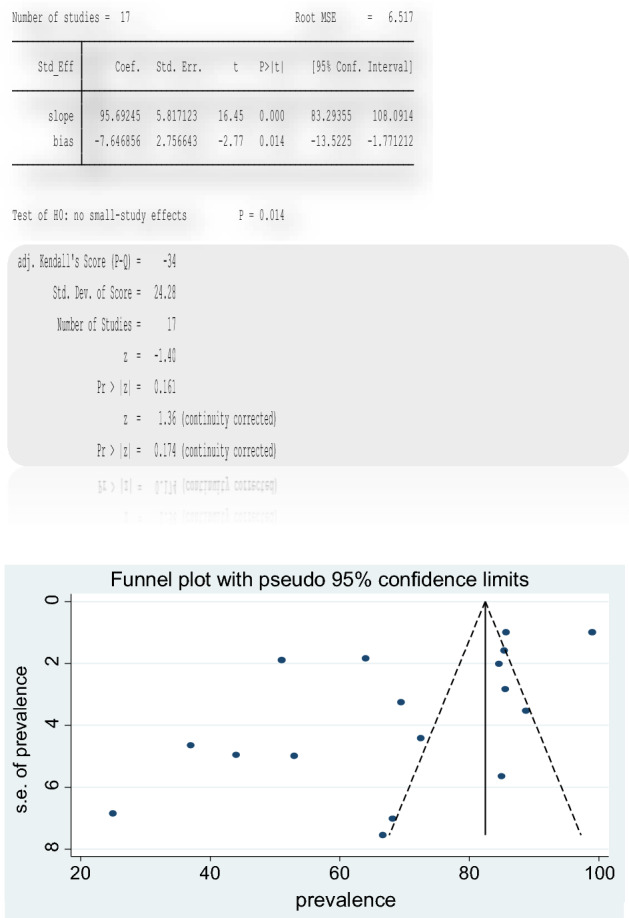
Funnel plot of included studies in the meta-analysis of the prevalence of anemia in TB.

Publication bias was assessed with a funnel plot and with visualization the graph of the funnel plot, there is asymmetry which suggests the presence of publication bias (Fig. 8). Egger's regression test (bias = − 7.65, p = 0.014) also demonstrated the existence of publication bias. However, begg’s test (z = 1.36, p = 0.174) suggested non-significant publication bias.

## Discussion

This current systemic review and meta-analysis showed the pooled prevalence of anemia, types of anemia, and morphological patterns among newly diagnosed people with TB in Africa. The pooled prevalence of anemia among people with TB was 69% in Africa which is higher than a finding of a systematic review and meta-analysis, 61.53%^11^. In addition, studies conducted in Korea, 37%^12^, Iran, 25.6%^34^, Russia, 36.5%^10^, and the Philippines, 32.4%^35^ reported lower prevalence of anemia. This finding is also higher that pooled prevalence of anemia among the general population^5^,and a study finding conducted in Ethiopia, (40.9%)^36^. However, studies conducted in Brazil, 89.2%^37^, and India, 100%^38^ reported significantly higher prevalence of anemia among people with TB. The observed variation could be attributed to the differences in the cut-off values used to diagnose anemia in the included studies. Additionally, it might be as a result of the differences in the characteristics of the study population included in the studies. In this meta-analysis, the heterogeneity between studies was substantial and may be attributable to variations among study population characteristics, such as sex, presence of HIV/AIDS.

The current meta-analysis shows that the prevalence of anemia of chronic disease among people with TB at diagnosis was 48%, while iron deficiency anemia was 11%. These findings are lower than a study done in Brazil, 75.9%^37^, and a systematic review and meta-analysis which reported a 20% of iron deficiency anemia among people with TB^11^.

In this review, 34% of people with TB had developed mild anemia at diagnosis. However, this rate of mild anemia is lower than studies reported from India, 60.8%^13^, and Korea, 84%^12^. A systematic review and meta-analysis also reported a higher prevalence of mild anemia among people with TB, 35.67%^11^.

The present review shows that normocytic normochromic anemia is the commonest morphological pattern of anemia, 32%. However, other studies have found microcytic hypochromic anemia to be the profound morphological pattern of anemia at TB diagnosis^39,40^.

Female population with TB had higher prevalence of anemia than male population with TB (74% vs. 67%). This result is supported by a finding from a systematic review and meta-analysis^11^. This finding was also noted in a systematic analysis of global anemia burden from 1990 to 2010, which showed that females had higher prevalence of anemia than males especially Central Asia (43.2% vs. 22.8%) and Asia Pacific (19.4% vs. 10%)^4^. A study conducted in India^41^ also indicated a more pronounce prevalence of anemia among females. The observed high rates of anemia among female TB patients as compared to males could be attributed to the existing differences in the physiological state of females and males which is related to the monthly loss of blood during menstruation^42^. In addition, the dietary habit of women in the region may contribute for the observed difference of the prevalence anemia among sex. This difference can be attributed to the variation of health seeking behaviour among males and females.

It is noted that the prevalence of anemia varies in the primary studies and the variation of the population included in these studies in terms of sample size, TB type, and co-morbidity presented and the definition of anemia significantly affected the prevalence of anemia among included studies and the observed heterogeneity showed in the pooled analysis of the studies was attributed with aforementioned reasons. Moreover, it should be noted that the observed inconsistences in the magnitude of the anemia in people with TB varies according to social, economic, lifestyle, cultures, presence of infectious diseases and health-seeking behaviors in different geographical areas. A previous study also reported a variation in growth of population, geographic area can attributed for some increment in the prevalence of anemia among people with TB^4^. Additionally, a study done in India^41^ revealed a high prevalence of anemia in people with low socioeconomic status, and low body weight.

Understanding of the high prevalence of anemia among people with TB from this meta-analysis has a great importance to conclude that anemia is a common hematologic disorder among people with TB in Africa, which can negatively influence the treatment outcome. Therefore, TB care and treatment interventions should consider mitigating the adverse consequences of anemia on people with TB such as death by instituting routine anemia screening at TB diagnosis.

The notable strength of this review is that studies were included in the meta-analysis after thorough quality assessment. However, the study is subjected to limitations. First, the measurement of anemia and the levels of anemia are inconsistent across the studies; the hemoglobin cut-off values for defining the outcomes are not based on the WHO criteria. Therefore, the outcome measurement may be over- or underestimated among the included studies. Second, the use of different study populations could contribute to the varied prevalence of anemia among studies which could results in a potential publication bias. Third, the searches have concentrated on a limited number of repertories of journals and grey literature sources and relevant articles might be omitted from the review. The inclusion of small sample studies in the meta-analysis resulted in almost equal weight for small studies and large studies. These findings might be undesirable in meta-analysis and could be due to the small studies might be poor quality. And the methodological error related to the use of random effects model in the meta-analysis might contribute for the substantive conclusion of resulting similar weight for small studies and large studies.

HIV status of study participants were not included among most studies reviewed for the meta-analysis and due to lack of data on the HIV status of participants’ sub-group analysis was not done to determine the effects of HIV status on the heterogeneity of studies. Lastly, the funnel plot suggests the likelihood of publication bias then, our results must be interpreted with caution.

In conclusion, despite the aforementioned limitations, the review indicates a high prevalence of anemia among people with TB in Africa. Anemia is frequently noted in female TB population than males at TB diagnosis. Whereas, the commonest types of anemia that presented at the time of TB diagnosis are anemia of chronic disease, mild and normochromic normocytic anemia. Hence, it is recommended to institute the routine screening of anemia at TB diagnosis and follow up to improve future treatment outcomes.

## Methods

### Literature search strategies

In this systematic review and meta-analysis, the preferred reporting items for systematic review and meta-analysis (PRISMA) guidelines were used^26^. We searched Medline/PubMed, Cochrane library, Science Direct, JBI database, the web of science, Google Scholar, WorldCat, Open Grey, Scopus, Agency for Healthcare Research and Quality, ProQuest, and African Journals Online to include both published articles and grey literature. The following terms, “tuberculosis,” “anemia,” “anaemia,” iron deficiency,” “hematological abnormality,” “haematological abnormality”, “micronutrient deficiency” were employed in the electronic search. The reference lists of included articles were also hand searched.

### Eligibility criteria

Studies that fulfilled inclusion criteria such as studies reported the prevalence of anemia among people with TB ≥ 15 years-old, both pulmonary and extra-pulmonary TB population with anemia, People with TB with or without HIV, clinical trials, cohort studies, case–control studies, cross-sectional studies, written in English, and conducted in Africa were included in the current review.

Studies were excluded if they were case reports, case series, commentaries, systematic reviews, and meta-analyses, non-English language publications, and reported prevalence of anemia among people with TB after initiation of anti-TB treatment, and retreatment cases.

### Study selection procedure

The search included articles published from April 2000 to December 2021 published in English language. The diagnosis of anemia was done by studies and studies were included in the analysis as long as they included the classification of anemia irrespective of the criteria used to diagnose anemia. The search was done between April and October 2021. The study selection was performed by two reviewers to determine which studies are suitable for systematic review and meta-analysis. Duplicated studies were excluded. The two reviewers independently screened articles as per the specified inclusion criteria. Disagreements between the two reviewers were resolved with discussions. Eligible studies were extracted by reviewing full texts. All studies that met the inclusion criteria were included in the final analysis.

### Data abstraction process

Two independent reviewers (YA & MS) extracted and recorded data from all included studies using predesigned abstraction checklists prepared in Microsoft Excel Spreadsheet. The data extracted included: author’s name, country, publication year, population, forms of TB, study design, sample size, anemia, levels of anemia, and morphological patterns of anemia, definitions, and measurement of anemia. Disagreements between reviewers were resolved with discussions.

### Quality assessment

The methodological quality of included studies were assessed based on standardized critical appraisal instruments from the Joanna Briggs Institute Meta-Analysis of Statistics Assessment and Review Instrument (JBI-MAStARI)^43^.

### Data synthesis

The data extracted from included studies were recorded in Microsoft Excel spreadsheet and were exported to STATA version 14 for further analysis. A random-effects model was used to calculate the pooled estimate with 95% CI. DerSimonian and Laird method was used as a variance estimator. Studies with small sample size were included in the meta-analysis. The meta-analysis used random effects model and there is substantial heterogeneity then the weights tend to become more equal. In this case a small study may have almost the same weight as a large one. Heterogeneity among studies was assessed with the I^2^ test statistic. Subgroup analysis was done based on sex category. Publication bias was assessed with funnel plot, and egger’s regression test. A p-value of less than 0.05 was used to declare the presence of publication bias.

## Data Availability

All relevant data are addressed in the manuscript, and additional data can be obtained upon reasonable request from the corresponding author.
